# Abstract of the Proceedings of the Buffalo Medical Association

**Published:** 1863-05

**Authors:** J. F. Miner

**Affiliations:** Secretary


ART. III.—*Abstract of the Proceedings of the Buffalo Medical Association.*

TUESDAY EVENING, April 7th, 1863.

This being the Annual Meeting, choice of officers resulted in the election of

| | |
|---|---|
| Dr. H. M. CONGAR, | President. |
| Dr. J. B. SAMO, | Vice President. |
| Dr. J. F. Miner, | Secretary. |
| Dr. C. C. WYCKOFF, | Treasurer. |
| Dr. J. B. SAMO, | Librarian. |

*For Primary Board*—Dr. SANDFORD EASTMAN, Dr. HENRY NICHELL, Dr. M. SHAW.

*Voted*, that the President be authorized to give credentials to any members of the Association who might signify a willingness to attend and represent the Society in the American Medical Association to meet in Chicago in June next.

*Voted*, James S. Smith, M. D., (House Physician and Surgeon of the Buffalo General Hospital,) a member of the Association, on compliance with the by-laws.

*Dr. Lockwood*, Chairman of the Committee appointed to revise the fee-bill for medical purposes, read the following report:

Your Committee to whom was entrusted the subject of changing the medical fee-bill, after a careful consideration of the question, have concluded to report in favor of changing the fee for each visit from $1 to $1.50, and for office prescription from 50 cents to $1.00. This advance the committee deem abundantly justified by the present depreciation of the currency and proportionate rise in all the necessaries of life. In other respects we are of opinion that the present fee-bill allows sufficient limit for advance in like proportion without making any essential change.

T. T. Lockwood,
James P. White.

This report was very fully and thoroughly discussed, most of the members present speaking to the points of interest, and all agreeing as to the propriety and necessity of increase of charge to meet the depreciated value of money or the increased value of every necessary of life. The importance of some uniform standard, below which it shall be regarded as dishonorable for a physician to make charge, to those who are able to pay, was lengthily insisted upon, and the practice, said to be common with some practitioners, of accepting business with the distinct understanding by the patient that not more than one-half the usual charge was to be made, was denounced as unprofessional and unfair.

The propriety of attending the poor, at such prices as their circumstances would require, was also admitted and advocated, and the unanimous opinion expressed, that this class of patients should not be distressed by physician's bills during his life or by their collection after his death.

The following resolution was offered by Dr. Strong and adopted:

*Resolved*, as the sense of this Society, that its members are in honor bound not to charge less than the minimum of the fee-bill.

*Voted*, that visits, examination with speculum, and applications, be charged from three to five dollars; office attendance of the same, from two to five dollars.

*Dr. Boardman* thought it wrong to enter charges upon our books greater in amount than would be right to have collected, since we might not be able to control our books. When physicians know the circumstances of patients, charge should be made of *only* the amount which he regarded proper to receive or have collected; the uncertainty of life rendered this important.

*Dr. Rochester*, at the suggestion of Dr. Nichell, spoke of the impossibility of the German physicians living strictly to the fee-bill, Dr. Nichell suggesting that the effect would be, to drive many families to the irregular German practitioners who are so numerous; and that at present it would be impossible for them to adhere strictly to the prices of the Society's fee-bill, Dr. Rochester admitting the force of Dr. Nicheli's suggestion, yet held that much could be done for their advantage, if they were united in their efforts, and resolutely insisted upon proper remuneration for their services.

*Voted*, that the Treasurer at the next meeting furnish the names of the members who have paid their dues, and of all actual members of the Association.

The Treasurer's Report was read, accepted, and voted to be placed on file.

*Dr. Miner* presented a diseased knee-joint for which he had the day previous made amputation. The patient was a young man, 19 years old, without any hereditary tendency to disease. Four years since, after a fall, his knee commenced to enlarge, and became very painful; for some months its use was suspended, and he was nearly or quite confined to his bed.— After this it grew gradually better, and he again regained the use of his knee nearly as perfect as ever. About two years since, while yet able to walk, run and jump, without apparent lameness, Dr. M. was consulted by the young man with view of operative interference to relieve the occasional pain, stiffness of the joint and enlargement of the knee. The case at that time was dismissed with advice "to let it alone." This was quite faithfully followed and he afterwards enjoyed great freedom from pain and complete use of his leg.

About six months since he fell in attempting to get upon a wagon, striking his knee upon the sharp edge of a board. After this accident he suffered great pain, the leg became strongly flexed upon the thigh, and the pain on the slightest motion was intolerable; emaciation became extreme, with loss of appetite and great prostration. With anodynes to allay the pain, stimulants and tonics, the boy rallied somewhat, and not being able to move a particle from his position in bed, and seeing no way of relief other than from amputation, he became very anxious to take its dangers and uncertainties, with full knowledge of all the facts.*

The sense of fluctuation was not distinct, and there was some reason to doubt that suppuration had taken place in the joint; yet there appeared no ground of expectation that recovery would take place, or that life even could be continued for any great length of time without amputation.

---

* This patient has recovered without accident of any sort, and is out upon his crutches.

The reason of presenting the morbid specimen to the Society was partly to show how at least in this instance, the disease was at the point of greatest pressure, the lower articulating surfaces being deeply ulcerated, apparently from the severe pressure induced by the flexion; and also to show how that extension of the leg while it would not relieve the amount of pressure would yet have placed two healthy surfaces in contact with each other, and might in this way have relieved the pain, while pressure might have been increased.

It will be observed that the under surface of the patella is also ulcerated and roughened, as is believed from the great pressure induced by the strong flexion of the leg. The specimen is an interesting one as showing the character and progress of scrofulous ulceration of cartilage and bone, in disease of the knee joint.

*Dr. Miner* also presented a specimen of rare disease as found in the testicle, which he had removed a few days since. It consisted of fibrous degeneration with cystic formation. It had been removed from a young man 19 years of age, and had been noticed by him for four or five years, producing no pain, but gradually increasing in size, until when removed it would weigh from twelve ounces to a pound. The different cysts contained fluid of different colors and density, the larger being filled with serous fluid containing a great amount of pigment, giving it a very black color, while others contained thick gelatinous substance, in some cases clear and transparent, in others yellow or opake. The distension of these fibrous cysts was very great; before puncture the cyst was hard and unyielding, distended to its utmost capacity. The walls of the cysts in some cases when distended, did not exceed a line in thickness, but when relieved of the contents contracted down, leaving no appearance of a cavity, and the walls three-fourths of an inch in thickness, showing the fibrous layers of the cyst, which were numerous and exceedingly elastic.

This form of disease must be much more rare in the testicle than ovary. Unilocular and multilocular—simple or compound cystic growth of the ovary being much more frequently met in practice than similar disease of the testicle. This patient had been under the care or been advised by three of the most experienced and capable physicians of Western New York, Drs. Poole, Emmons and Colegrove, the two former assisting in its removal and subsequent examination, who regarded its appearance in the testicle as rare.

There can be little if any danger of a return of the disease, and the patient as he had since learned from Dr. Poole, had recovered rapidly from the operation. Dr. Poole had also informed him that "the patient at birth had a twin sister; had always himself been delicate; and had breasts developed as fully as a girl at puberty; this accounting possibly for lack of development in the other testicle and in the penis, or at least completing more perfectly the history of the disease." The nature of this disease could hardly be regarded doubtful. It could only be confounded with cystic saracoma, and does not resemble it when the history and complications of malignant disease are remembered.

*Dr. Eastman* presented a small growth which he thought was an epithelial cancer, which he had removed from the temporal region, including with the growth, the neighboring tissue, and hoped by this means to prevent its re-appearance. It first attracted attention about six months previous to removal.

*Voted*, to adjourn to the first Tuesday evening in May.

J. F. Miner, *Secretary.*

